# Arabidopsis mTERF15 Is Required for Mitochondrial *nad2* Intron 3 Splicing and Functional Complex I Activity

**DOI:** 10.1371/journal.pone.0112360

**Published:** 2014-11-17

**Authors:** Ya-Wen Hsu, Huei-Jing Wang, Ming-Hsiun Hsieh, Hsu-Liang Hsieh, Guang-Yuh Jauh

**Affiliations:** 1 Institute of Plant Biology, National Taiwan University, Taipei, 116, Taiwan, ROC; 2 Institute of Plant and Microbial Biology, Academia Sinica, Nankang, Taipei, 11529, Taiwan, ROC; 3 Biotechnology Center, National Chung-Hsing University, Taichung, 402, Taiwan, ROC; International Centre for Genetic Engineering and Biotechnology, Italy

## Abstract

Mitochondria play a pivotal role in most eukaryotic cells, as they are responsible for the generation of energy and diverse metabolic intermediates for many cellular events. During endosymbiosis, approximately 99% of the genes encoded by the mitochondrial genome were transferred into the host nucleus, and mitochondria import more than 1000 nuclear-encoded proteins from the cytosol to maintain structural integrity and fundamental functions, including DNA replication, mRNA transcription and RNA metabolism of dozens of mitochondrial genes. In metazoans, a family of nuclear-encoded proteins called the mitochondrial transcription termination factors (mTERFs) regulates mitochondrial transcription, including transcriptional termination and initiation, via their DNA-binding activities, and the dysfunction of individual mTERF members causes severe developmental defects. *Arabidopsis thaliana* and *Oryza sativa* contain 35 and 48 mTERFs, respectively, but the biological functions of only a few of these proteins have been explored. Here, we investigated the biological role and molecular mechanism of Arabidopsis mTERF15 in plant organelle metabolism using molecular genetics, cytological and biochemical approaches. The null homozygous T-DNA mutant of *mTERF15*, *mterf15*, was found to result in substantial retardation of both vegetative and reproductive development, which was fully complemented by the wild-type genomic sequence. Surprisingly, mitochondria-localized mTERF15 lacks obvious DNA-binding activity but processes mitochondrial *nad2* intron 3 splicing through its RNA-binding ability. Impairment of this splicing event not only disrupted mitochondrial structure but also abolished the activity of mitochondrial respiratory chain complex I. These effects are in agreement with the severe phenotype of the *mterf15* homozygous mutant. Our study suggests that Arabidopsis mTERF15 functions as a splicing factor for *nad2* intron 3 splicing in mitochondria, which is essential for normal plant growth and development.

## Introduction

Mitochondria, which originated through the endosymbiosis of α-proteobacteria into ancestral host cells, are the cellular powerhouses and play vital roles in diverse eukaryotic cell processes through the production of ATP and various metabolic intermediates [1], [2]. Recent studies also suggest that dysfunctional mitochondria are involved in many neurodegenerative diseases such as aging and cognitive decline in a wide range of metazoans, including humans [3]. Maintaining the structural and metabolic integrity of this semi-autonomous organelle is essential for the normal function of eukaryotic cells. Nevertheless, over the course of symbiotic evolution, the majority of mitochondrial genes migrated into the nuclear genome of the original host, leaving an incomplete set of essential genes in the mitochondrial genomes of most organisms, including plants [4]–[6]. Complicated and dynamic communication and coordination between the nucleus and mitochondria greatly impact many fundamental cellular processes in, and even the lives of, most eukaryotes [7]. Indeed, based on the complete sequence of the Arabidopsis mitochondrial genome, it has been reported that 57 mitochondrial genes encode the subunits of multiprotein complexes that are required for the respiratory chain, heme and cytochrome assembly, and mitochondrial ribosomes [4]. Additionally, plant mitochondria are more complex than those found in other kingdoms and exhibit unique RNA metabolism, including RNA transcription, splicing, editing, degradation, and translation [8]–[10]. The proteins involved in these processes are predominantly encoded by the nuclear genome and are imported into mitochondria after protein synthesis. For example, recent studies suggested that one protein family, called the mitochondrial transcription termination factors (mTERFs), plays important roles in regulating the organellar transcription machinery.

The mTERF proteins were first identified two decades ago as regulators of transcription termination in human mitochondria [11]. Phylogenetic analyses of mTERF homologs in metazoans and plants revealed the presence of 4 subfamilies, mTERF1 to mTERF4 [12]. These proteins share a common 30-amino-acid repeat module called the mTERF motif [13]. The proteins within this family possess diverse numbers and arrangements of these motifs, yet the folding patterns of these proteins are similar. Moreover, crystal structure studies of mTERF1, mTERF3 and mTERF4 suggest that the helical structure of the mTERF motifs may be essential for their nucleic acid-binding activities [14], [15]. Human mTERF1 binds specific sites located at the 3′-end of the 16S rRNA and tRNA^Ler(UUR)^ genes to terminate mitochondrial transcription [16]. Additionally, mTERF1 binds to the mitochondrial transcription initiation site to create a DNA loop that allows for the recycling of the transcriptional machinery [16]. This simultaneous link between mitochondrial transcriptional initiation and termination sites may explain the high rate of mitochondrial rRNA biogenesis. mTERF2 binds to mitochondrial DNA (mtDNA) in a non-specific manner and associates with nucleoids. *mTERF2* loss-of-function mice exhibit myopathies, memory deficits, and impaired respiratory function due to a reduction in the number of mitochondrial transcripts [17], [18]. *mTERF3* is essential, and mTERF3-knockout mice die during the early stages of embryogenesis; mTERF3 non-specifically interacts with mtDNA promoter regions and mediates the repression of mitochondrial transcription initiation [19]. Mouse NSUN4 (NOL1/NOP2/Sun domain family, member 4) is a methyltransferase involved in the methylation of ribosomes. Mouse mTERF4 first binds 16S and 12S rRNAs, then forms a stoichiometric complex with NSUN4, which is essential for proper mitochondrial ribosomal assembly and translation [14], [20], [21].

The genome of Arabidopsis contains 35 identified mTERFs, which vary in the number and arrangement of their mTERF motifs [22]. Several recently characterized Arabidopsis mTERF proteins displayed diverse and complicated functions during mitochondrial and plastid transcription, such as *SINGLET OXYGEN-LINKED DEATH ACTIVATOR10* (*SOLDAT10*) [23], *BELAYA SMERT*/*RUGOSA2* (*BSM/RUG2*) [22], [24], *MTERF DEFECTIVE IN ARABIDOPSIS 1* (*MDA1*) [25] and *SUPPRESSOR of hot1-4 1* (*SHOT1*) [26]. SOLDAT10, the first Arabidopsis mTERF to be characterized, is targeted to plastids and regulates plastid homeostasis. Arabidopsis *soldat10* mutants suppressed the ^1^O_2_-mediated cell-death and growth-retardation phenotypes of the conditional *fluorescent* (*flu*) mutant, in which excess levels of the reactive oxygen species (ROS) ^1^O_2_ were generated within chloroplasts after the dark-to-light shift [23]. BSM/RUG2 is essential for embryogenesis and postembryonic development in Arabidopsis and displays DNA-binding activity towards plastid DNA. Additionally, BSM/RUG2 localizes to both plastids and mitochondria, and the perturbation of BSM/RUG2 affects the processing and steady-state levels of plastid transcripts [22] and decreases the expression of mitochondrial transcripts [24]. Arabidopsis MDA1 is a plastid-localized protein involved in abiotic stress tolerance. *MDA1* mutation leads to significant defects in plant growth and chloroplast development but enhanced tolerance to salt and osmotic stress, perhaps via reduced sensitivity to abscisic acid [25]. Mutating *SHOT1* suppresses the heat-hypersensitive phenotype caused by the loss of heat shock protein 101 in Arabidopsis [26]. However, SHOT1 is a mitochondrial-resident mTERF protein, and the thermotolerance phenotype of *shot1* may be caused by reduced ROS-mediated oxidative damage during heat stress. Additionally, SHOT1 plays an important role in regulating the expression of mitochondrial-encoded genes and mitochondrial genome copy number. Although it is known that many mTERF mutations cause defective development and stress responses, studies of the precise molecular functions of these mTERF proteins are limited in Arabidopsis organelles.

In Arabidopsis, several protein families that participate in organellar RNA splicing, including those containing the chloroplast RNA splicing and ribosome maturation (CRM) domain [27]–[29], the pentatricopeptide repeat (PPR) [30]–[35], the plant organellar RNA recognition (PORR) domain [36], [37] and the ACCUMULATION OF PHOTOSYSTEM ONE (APO) motif [38]. These domains are recognized as RNA binding domains and interact with specific elements within introns. Although several splicing factors have been identified in plastids and mitochondria, the detailed mechanism of RNA splicing in plant organelles is still being investigated. Recently, maize mTERF4 was identified as a splicing factor associated with several introns and splicing factors in chloroplasts [39].

In the present study, we investigated another Arabidopsis mTERF protein, mTERF15, and identified its role in post-transcriptional modification of mitochondrial RNA processing. Mutating *mTERF15* significantly disturbed normal plant vegetative and reproductive growth, which may be the result of defective mitochondrial development and/or function. Interestingly, mTERF15 is a mitochondria-localized protein that binds to RNA but not to double-stranded DNA (dsDNA). Moreover, an analysis of mitochondrial intron splicing in *mterf15* mutants revealed defective RNA splicing of *nad2* intron 3, which led to a deficiency in complex I activity. The possible molecular function of mTERF15 in regulating Arabidopsis mitochondrial RNA metabolism is discussed.

## Materials and Methods

### Plant material and growth conditions

The *mTERF15* T-DNA insertion mutant (SALK_134099) was obtained from the Arabidopsis Biological Resource Center (Ohio State University, USA). Seeds from *Arabidopsis thaliana* ecotype Columbia-0 and *mterf15/+* plants were surface-sterilized by chloride gas after stratification for 3 d at 4°C and then either germinated on half-strength Murashige and Skoog medium (Duchefa Biocheme, The Netherlands) containing 1% sucrose and 0.7% phytoagar (Duchefa) or grown in soil inside a growth chamber on a 16-h light/8-h dark cycle at 22°C.

### Complementation of the *mterf15* mutant

Approximately 3.3 kb of the genomic sequence corresponding to locus *At1g74120*, including a 2-kb putative promoter and the 1.3-kb coding region, was amplified using Phusion polymerase (Finnzymes, http://www.finnzymes.com) and then ligated into a modified *pPZP221* vector containing a C-terminal GFP sequence followed by the Nopaline synthase (NOS) terminator. The primers used for cloning are in Table S1. After verification of the DNA sequence, the construct was introduced into *Agrobacterium tumefaciens* strain GV3101. Arabidopsis plants heterozygous for *mTERF15* were transfected with *Agrobacterium* by the vacuum infiltration method [40]. Seeds corresponding to the T_1_ generation were collected and grown on solid half-strength Murashige and Skoog medium (Duchefa) containing 1% sucrose, 0.7% phytoagar (Duchefa) and the antibiotic G418 at 100 µg/µl. Leaves from T_1_ plants were harvested for genotyping. Plants with an *mterf15* homozygous background harboring *mTEFR15p::mTERF15-GFP* were collected for further studies.

### Ultrastructural sample preparation and tetramethylrhodamine methyl ester perchlorate (TMRM) staining

For transmission electron microscopy, samples were frozen in a high-pressure freezer (Leica EMPACT2, 2000–2050 bar). Freeze substitution involved the use of anhydrous acetone (containing 1% OsO_4_ and 0.1% UA) with a Leica EM AFS2. The samples were kept at −85°C for 3 d, at −60°C for 1 d, at −20°C for 1 d, and at 0°C for 1 d and then brought to room temperature. The samples were infiltrated and embedded in Spurr resin. Ultrathin sections (70–90 nm) were cut using a Reichert Ultracut S or Leica EM UC6 (Leica, Vienna, Austria) and collected on 100 mesh copper grids. After staining with 5% uranyl acetate in 50% methanol for 10 min and 0.4% lead citrate for 4 min, sections were observed under a Philips CM 100 Transmission Electron Microscope at 80 KV; images were captured using a Gatan Orius CCD camera.

TMRM staining (Molecular Probes, Invitrogen) was performed to examine the mitochondrial membrane potential in protoplasts. Isolated protoplasts were stained with 20 nM TMRM and immediately observed under a laser scanning confocal microscope (Zeiss, LSM510, Carl Zeiss, http://www.zeiss.com). TMRM fluorescence and chlorophyll autofluorescence were induced by excitation with a 543-nm HeNe laser and a 488-nm Argon laser. The emission signals from TMRM and from chlorophyll were recorded with using 565- to 615- and 650-nm band-pass filters, respectively. The fluorescence intensity and area were quantified using LSM510 software (Carl Zeiss, http://www.zeiss.com).

### Northwestern blot analysis

Northwestern blot analysis was performed to test the RNA-binding ability of mTERF15 as described previously [41]. Briefly, *mTERF15* cDNA lacking the 34 N-terminal amino acids was ligated into the pET52b vector to produce the mTERF15-strep recombinant protein. The recombinant proteins were expressed in *E. coli* strain BL21 and incubated with StrepTactin-Sepharose resins (GE Healthcare) according to the manufacturer's instructions. The resins were washed extensively with incubation buffer before elution of the recombinant proteins. The mTERF15-strep recombinant protein and the MBP-strep control protein were transferred from 12% SDS-polyacrylamide gels onto a PVDF membrane and then renatured overnight in renaturation buffer (0.1 M Tris-HCl, pH 7.5 and 0.1% (v/v) NP-40) at 4°C. After 4 washes with renaturation buffer (15 min), blots were blocked with blocking buffer [(10 mM Tris-HCl, pH 7.5, 5 mM Mg acetate, 2 mM DTT, 5% (w/v) BSA and 0.01% (v/v) Triton X-100)] at room temperature for 5 min. Full length of *nad2* intron 3 was *in vitro* synthesized using SP6 polymerase (Promega). Total RNA and *in vitro* synthesized *nad2* intron3 were labeled with γ^32^P ATP using T4 polynucleotide kinase (Fermentas). Hybridization was performed at 4°C in the presence of 5 µg labeled RNA. Blots were washed 4 times (5 min each) in a washing buffer (10 mM Tris-HCl, pH 7.5, 5 mM Mg acetate, 2 mM DTT) to remove unbound RNA. The radioisotope signals were detected via autoradiography on X-ray film (Kodak).

### RNA isolation and qRT-PCR analysis

Total RNA was isolated using the Qiagen RNeasy Mini Kit (QIAGEN) and then treated with TURBO DNA-free DNase (Ambion) to remove contaminating genomic DNA. Reverse transcription of RNA was performed using the M-MLV transcriptase system (Invitrogen) and random hexamer primers.

Gene expression analysis via qRT-PCR was carried out using Power SYBR Green Supermix (Applied Biosystems) running on an ABI Prism 7000 sequence detection system (Applied Biosystems). Primer sets were designed as described previously [31] (Table S2) to monitor the expression of genes involved in the splicing of junctions within mitochondria.

### Northern blot analysis

A total amount of 5 µg RNA was denatured at 65°C for 10 min and then separated on 2% formaldehyde-agarose gels in MOPS buffer and transferred to a nylon membrane (PerkinElmer). The *nad2* probes were labeled using the Rediprime II DNA labeling system (GE Healthcare). Probe primers are listed in Table S1. The prehybridization, hybridization and washing steps were performed following the manufacturer's instructions.

### Crude mitochondria isolation and complex I Activity assay

Crude mitochondria were prepared as described previously [36], [42]. Approximately 200 mg fresh tissue was extracted in 2 ml extraction buffer (75 mM MOPS-KOH, pH 7.6, 0.6 M sucrose, 4 mM EDTA, 0.2% polyvinylpyrrolidone 40, 8 mM cysteine, 0.2% bovine serum albumin) at 4°C. The lysate was centrifuged first at 1300×g for 5 min (twice), after which the supernatant was centrifuged at 22,000×g for 20 min at 4°C. The resulting pellet (which usually contains most of the thylakoid and mitochondrial membranes) was resuspended in buffer (10 mM MOPS-KOH, pH 7.2, 0.3 M sucrose) and stored at −80°C.

Clear native electrophoresis was performed as described previously [43]. Crude mitochondria were washed with distilled water, resuspended with buffer (50 mM NaCl, 50 mM imidazole/HCl, 2 mM 6-aminohexanoic acid, 1 mM EDTA, pH 7.0) and solubilized by adding DDM (10%) at a DDM-to-protein ratio of 2.5 (g/g). After centrifugation at 100,000×g for 15 min, 10% glycerol and 0.02% Ponceau S were added to the supernatant for 4–16% native PAGE with anode buffer (25 mM imidazole/HCl, pH 7.0) and cathode buffer (50 mM Tricine, 7.5 mM imidazole, pH 7.0, 0.01% DDM and 0.05% DOC). Clear native gels were washed 3 times with distilled water for 5 min and incubated with 1 mM nitroblue tetrazolium and 0.14 mM NADH in 0.1 M Tris, pH 7.4. The reaction was stopped with 40% methanol and 10% acetic acid after a dark blue signal appeared on the gel.

## Results

### Disruption of the Arabidopsis *mTERF15* gene results in significant growth and developmental retardation

**Figure 1:**
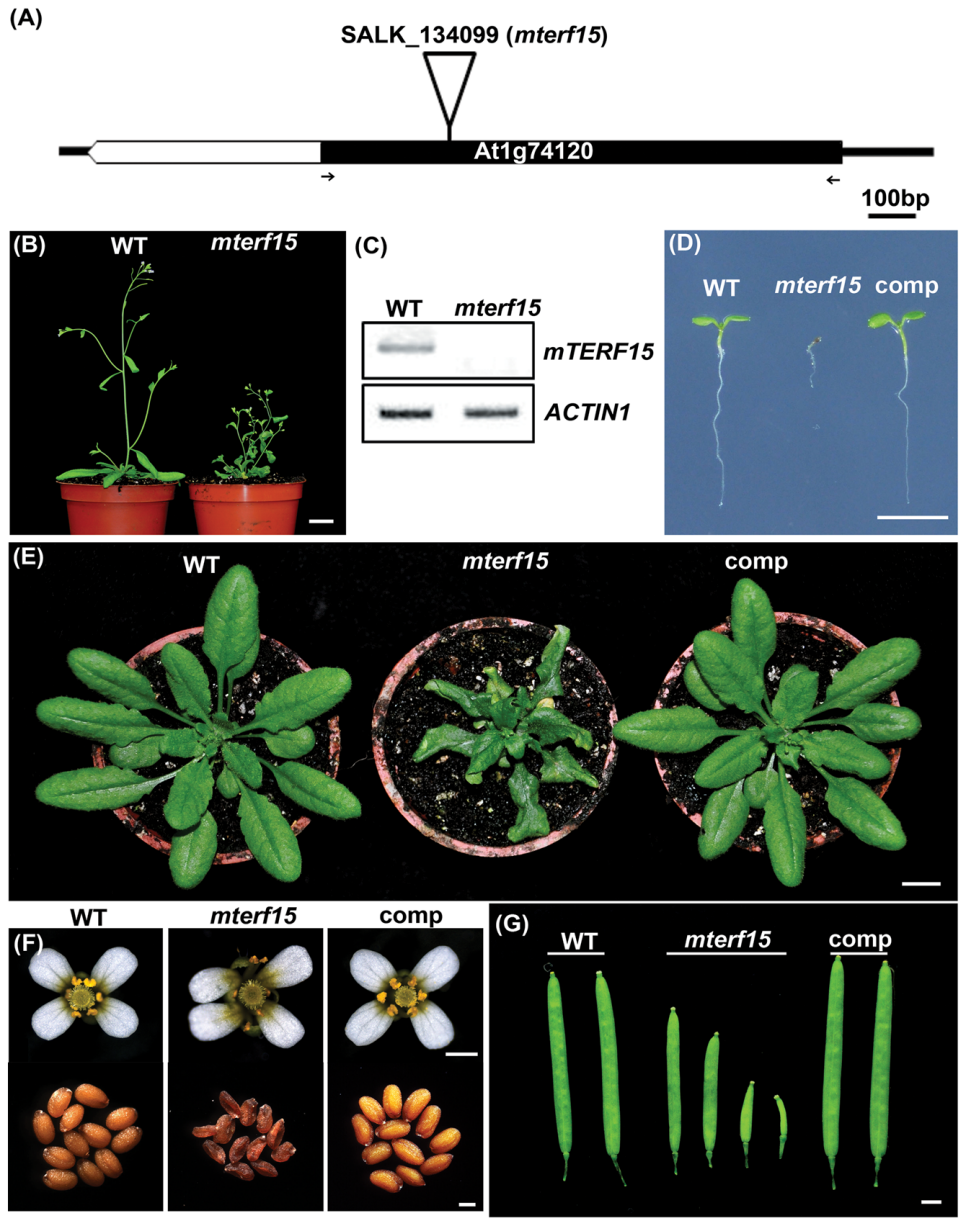
Phenotypes associated with *mterf15* mutants. (**A**) Schematic diagram of the Arabidopsis *mTERF15* gene (At1g74120) and the single T-DNA insertion within the coding region for *mterf15* (SALK_134099). Primers used to analyze *mTERF15* transcripts are indicated by black arrows. (**B**) Comparison of whole-plant morphology between a 1.5-month-old wild-type plant and a 3-month-old *mterf15* mutant. Scale bar  = 1 cm. (**C**) RT-PCR analysis of *mTERF15* expression in 7-d-old wild-type (WT) and *mterf15* seedlings. The housekeeping gene *ACTIN1* was used as an internal control. (**D**) Morphology of 7-d-old seedlings for wild-type and *mterf15* lines and a complemented line transformed with *mTERF15p::mTERF15-GFP*. Scale bar  = 1 cm. (**E**) Morphology and arrangement of leaves for 1-month-old wild-type, 2-month-old *mterf15* and 1-month-old *mterf15* complementation lines. Scale bars  = 1 cm (**F**) Morphology of flowers and seeds from the wild-type, *mterf15* and complementation lines. Scale bars  = 1 mm (flowers) and 200 µm (seeds). (**G**) Morphology of siliques from the wild-type, *mterf15* and complementation lines. Scale bars  = 1 mm.

A screen of Arabidopsis T-DNA insertional mutants from the SALK collection allowed us to identify several mutants that were defective in post-embryonic development and/or seed germination. We found one T-DNA mutant showing abnormal phenotype in progenies segregated from SALK_134099 heterozygotes. The progeny of SALK_134099 heterozygotes showed growth retardation at a ratio of 3∶1 (normal to defective) (χ^2^, *p* = 0.924; Table 1). Most abnormal progenies derived from SALK_134099 heterozygous plants were unable to germinate on solid medium (17.02%), although a few grew slowly (8.21%, Table 1). The SALK_134099 homozygous mutant containing a T-DNA insertion in the coding region of Arabidopsis *mTERF15* (At1g74120; Figure 1A) and showing significant growth and development retardation was named *mterf15* (Figure 1B). The full-length *mTERF15* transcript was not detected in the homozygous *mterf15* plant (Figure 1C), suggesting that all of the observed phenotypes resulted from the null mutation of the *mTERF15* allele (SALK_134099). Developmental defects were widespread in homozygous *mterf15* plants. Compared with wild-type seedlings, *mterf15* seedlings were small in stature, had small cotyledons and displayed reduced growth of primary roots (Figure 1D). The rosette leaves of *mterf15* were smaller, wrinkled and twisted whereas the wild type plant showed larger and smooth round-shaped leaves. Moreover, the extended petiole region of *mterf15* was shorter than the wild type plant. Compared with 1.5-month-old wild-type plants that were ready for blossoming, 3-month-old adult *mterf15* plants were dwarfed and just about ready for flowering (Figure 1E). The flowers of *mterf15* plants were abnormal, with misplaced petals, extremely defective anthers and a limited amount of pollen (Figure 1F, upper panel). Unlike wild-type siliques, mature *mterf15* siliques were much shorter (Figure 1G), and no viable seeds were found (Figure 1F, lower panel).

**Table 1:** The phenotype of progeny from SALK 134099 heterozygotes (*mterf15/+*) with abnormal morphology.

	Normal seedlings	Seedlings with retarded growth	Non-germinated seeds	Ratio (normal: abnormal)	*p*-value (χ^2^ value, 3∶1)
**Wild-type**	**308 (99.68)**	**0**	**1 (0.32)**	* **NA**	**NA**
**SALK_134099 heterozygotes**	**246 (74.77)**	**27 (8.21)**	**56 (17.02)**	**2.96∶1**	**0.924**

Phenotypes were recorded after seeds were germinated on half-strength MS solid medium and then incubated for 7 d with a 16-h light/8-h dark cycle at 22°C.

*NA, Not available.

Data indicates total number of plants observed and numbers in brackets indicate their corresponding percentage.

To further verify that these defects were indeed caused by the disruption of the *mTERF15* allele, *mterf15* heterozygotes were complemented with the *mTERF15* native promoter-driven *mTERF15* coding sequence tagged with *GFP* (*mTERF15p::mTERF15-GFP*). The T_2_ progeny of homozygotes for the *mTERF15* mutation which harbors at least one copy of the transgene *mTERF15p::mTERF15-GFP* showed similar phenotypes as 7-d-old wild-type seedlings and adult plants (Figure 1 and Figure S1). All of the above results show that functional *mTERF15* is essential for normal plant growth and development.

### mTERF15, a plant-specific and mitochondria-localized protein, is important for mitochondrial biogenesis and membrane potential

The Arabidopsis *mTERF15* locus contains a single exon and appears to encode a 51-kDa protein. Architecture analysis with SMART (http://smart.embl-heidelberg.de) revealed that mTERF15 contains 5 typical mTERF motifs (Figure S2A). A search of the NCBI database (http://www.ncbi.nlm.nih.gov) revealed mTERF15 homologs present in *Vitis vinifera*, *Populus trichocarpa*, *Brachypodium distachyon*, *Sorghum bicolor* and *Zea mays*. Comparing protein similarities and identities between flowering plant mTERF15 orthologs and metazoan mTERF1, Arabidopsis mTERF15 is evolutionally closer to plant mTERF15 orthologs than metazoan mTERF1. This result suggests that mTERF15 is a plant-specific mTERF protein (Table S3). The phylogenetic tree branched into 2 groups, monocots and dicots (Figure S2B). Amino acid sequence alignments of all 6 homologs revealed stronger conservation of the C terminus compared with the N terminus. All of these homologs appeared to contain 5 mTERF motifs with a conserved proline residue at position 8, except for the first motif (Figure S2A).

*In silico* analysis of the BIO-array (http://bar.utoronto.ca/efp_arabidopsis/cgi-bin/efpWeb.cgi) and Genevestigator (https://www.genevestigator.com/gv/plant.jsp) microarray databases suggested various expression levels of *mTERF15* in different tissues, with the highest and lowest expression levels in mature seeds and pollen, respectively. However, in the Genevestigator microarray database, *mTERF15* showed the highest expression in sperm cells and the lowest in leaf phloem protoplasts. Quantitative real-time PCR spatiotemporal analysis in Arabidopsis revealed *mTERF15* expression in all organs, with high levels of accumulation in seedlings, flowers and siliques (Figure S3).

Transient protoplast assays and stable transgenic studies suggested that mTERF15 is a mitochondrial protein [22]. To confirm the subcellular localization of mTERF15, we observed the root hairs of 7-d-old *mTERF15p::mTERF15- GFP* transgenic seedlings via confocal microscopy after staining with the mitochondrial indicator MitoTracker orange. The co-localization of the mTERF15-GFP and MitoTracker signals demonstrated that mTERF15 is a mitochondrial protein (Figure S4).

**Figure 2:**
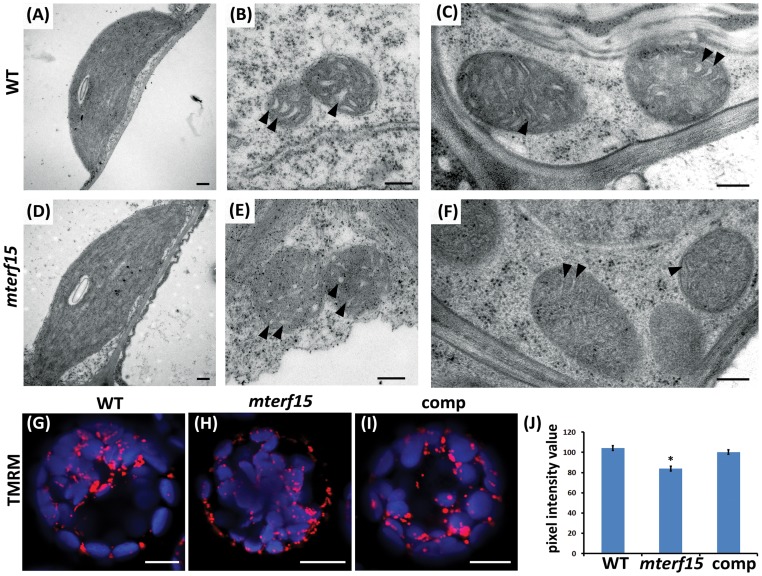
Morphological analysis of mitochondria in wild-type (WT) and *mterf15* plants. (**A** and **D**) Transmission electron microscopy of thin sections corresponding to chloroplasts from 1-month-old wild-type (**A**) and 2-month-old *mterf15* plants (**D**). Sections were processed by chemical fixation. Scale bar  = 2 µm. (**B**, **C**, **E**, **F**) Transmission electron microscopy of thin sections corresponding to mitochondria from wild-type (**B**, **C**) and *mterf15* (**E**, **F**) plants. Images B and E correspond to chemically fixed leaves. Images C and F correspond to leaves fixed by high-pressure freezing. Scale bar  = 1 µm. Arrowheads pointed the inner membrane spaces in mitochondria. (**G**, **H**, **I**) Overlay of signals from chlorophyll autofluorescence (blue) with signals from the mitochondrial membrane potential marker tetramethylrhodamine methyl ester perchlorate (TMRM; red) in protoplasts from the wild-type (**G**), *mterf15* (**H**) and complementation lines (**I**). Scale bar  = 10 µm. (**J**) Quantification of the mean fluorescent intensity after TMRM staining. Data are mean ± SD of 15 individual protoplasts from the wild-type, *mterf15* and complementation lines. Asterisks represent significant differences (*P<0.5, **P<0.01, ***P<0.001; Student's *t* test) relative to wild type.

We further investigated the morphology of chloroplasts and mitochondria from the leaves of 1-month-old wild-type and 2-month-old *mterf15* plants by transmission electron microscopy. The sizes and features of *mterf15* chloroplasts were similar to those of wild-type chloroplasts (Figure 2A, D). Although the sizes of the mitochondria did not differ between *mterf15* and wild-type plants, the inner membrane systems were aberrant in *mterf15* mitochondria; wild-type mitochondria (Figure 2B–2C) showed normal cristae structures with clear inner spaces, whereas *mterf15* mitochondria (Figure 2E–2F) lacked any obvious cristae structure. Therefore, the integrity of the mitochondrial inner membrane system may have been compromised, potentially resulting in decreased mitochondrial membrane potential.

To assess mitochondrial membrane integrity, we treated protoplasts from leaves of the wild-type, complementation line and *mterf15* plants with tetramethylrhodamine methyl ester (TMRM), a lipophilic, cationic, red-orange fluorescent dye that accumulates within mitochondria in living cells. The intensity of fluorescence is associated with the mitochondrial membrane potential [44], [45]. We observed large and strong fluorescent TMRM signals in protoplasts from the wild-type and complementation lines, which suggests that mitochondria were active in these lines (Figure 2G, I); however, TMRM signals were slightly weaker in *mterf15* protoplasts (Figure 2H). The mean fluorescence intensity for *mterf15* protoplasts was reduced by ∼80% compared with wild-type protoplasts (Figure 2J). These results suggest that mTERF15 is crucial for maintaining mitochondrial biogenesis and membrane integrity.

### mTERF15 is an RNA-binding protein that is involved in mitochondrial *nad2* intron 3 splicing and normal complex I activity

**Figure 3:**
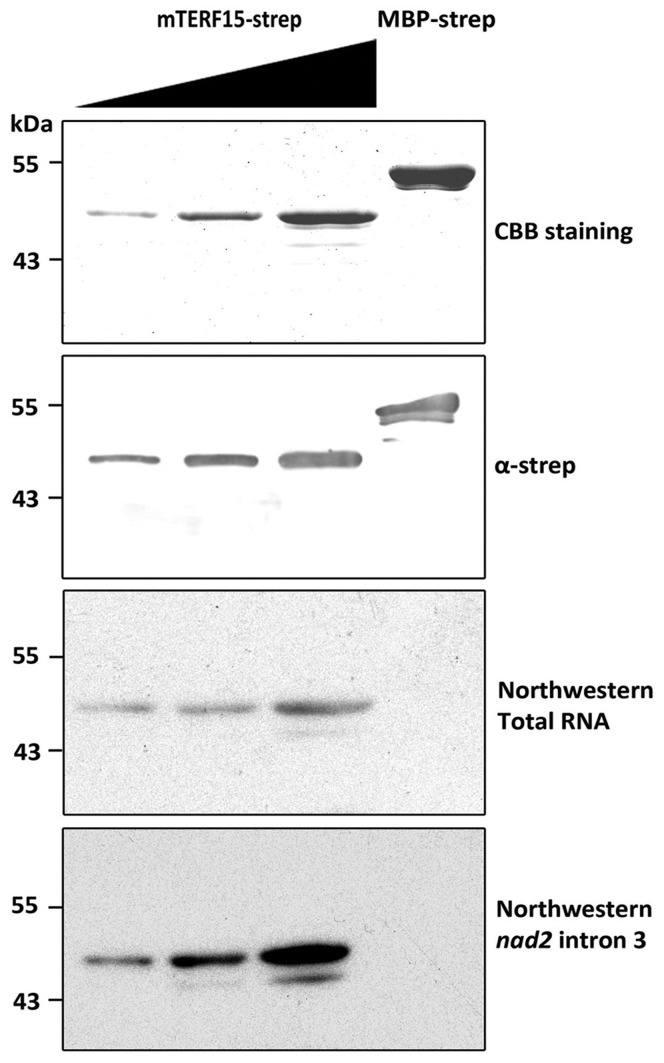
Northwestern blot assay of RNA-binding by mTERF15 recombinant protein. The first panel shows Coomassie brilliant blue (CBB) staining of MBP-strep and mTERF15-strep lacking the first 34 N-terminal amino acids. The second panel shows immunoblot analysis with anti-strep antibody to recognize the strep-tag. The third panel shows the northwestern blot analysis using radioisotope-labeled total RNA isolated from 7-d-old seedlings. The final panel shows the northwestern blot analysis using radioisotope-labeled *nad2* intron 3; signals are observed on the PVDF membrane corresponding to the RNAs bound to mTERF15.

Because most eukaryotic mTERFs function as nucleic acid-binding proteins, we performed *in vitro* binding studies with dsDNA-cellulose and northwestern blot analyses to determine the dsDNA- and RNA-binding abilities of mTERF15. After incubation with commercial dsDNA-cellulose resin, most of the mTERF15 was present in the unbound fraction (Figure S5A), which implies a lack of dsDNA-binding activity for mTERF15. Additionally, southwestern blot analysis was employed to validate the DNA-binding ability of mTERF15. As shown in Figure S5B, there is no DNA-binding signal for the mTERF15 protein in southwestern blots. The above results imply that mTERF15 may not be a DNA-binding protein. Therefore, we investigated the RNA-binding ability of mTERF15 by northwestern blot analysis (Figure 3). Strep-tagged mTERF15 lacking the first N-terminal 34 amino acids, which contain a putative targeting signal (MW  = 48 kDa), was separated by SDS-PAGE, transferred to a PVDF membrane and probed with a specific antibody against strep-tagged or radiolabeled total RNA prepared from 7-d-old Arabidopsis seedlings. The presence of the mTERF15-strep and MBP-strep fusion protein were confirmed by immunoblot analysis using an antibody against the strep-tag, and 48-kDa bands corresponding to mTERF15-strep were detected in a dose-dependent manner in the RNA-binding assay but not in the control. These results suggest that Arabidopsis mTERF15 functions as an RNA-binding protein.

**Figure 4:**
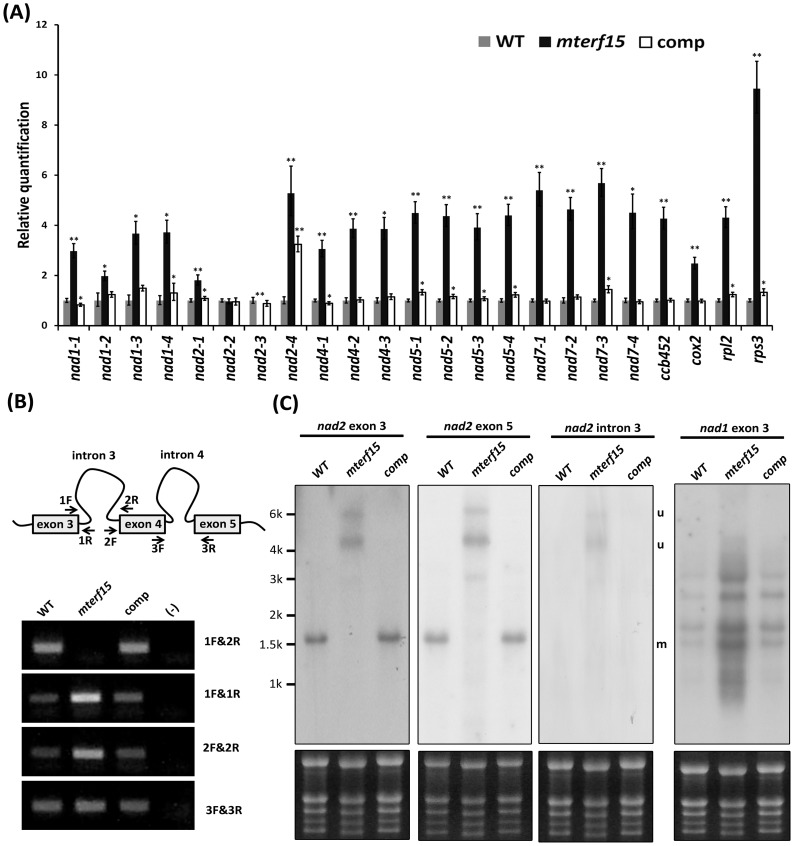
Mitochondrial splicing efficiency in *mterf15* mutants. (**A**) qRT-PCR analysis of mitochondrial RNA splicing in 7-d-old seedlings from the wild-type, *mterf15* and complementation lines. Data were normalized to the expression of 5 housekeeping genes (*YSL8*, *RPL5B*, *UBC*, *ACTIN2/ACTIN8* and *TUB6*). Asterisks represent significant differences (*P<0.5, **P<0.01; Student's *t* test) relative to wild type. (**B**) Diagram of *nad2b* transcripts including exon 3 to 5 and RT-PCR analysis of transcript levels from the *nad2* exon–intron junction. Primers used for RT-PCR analysis are indicated with black arrows. (**C**) Northern blot analysis using probes recognizing *nad2b* transcripts (*nad2* exon 3 and *nad2* exon 5), *nad2* intron 3, and *nad1* exon 3. The radioisotope signal is shown in the top panel, and the rRNA signals shown in the lower panel were used as loading controls. Marker sizes are indicated. m, mature transcript; u, un-spliced transcript.

Previously, Babiychuk *et al*. (2011) showed that one mTERF protein, BSM, is required for embryogenesis and plays a role in chloroplast intron splicing. Therefore, we examined 23 intron-splicing events in mitochondria by quantitative RT-PCR (qRT-PCR). In mitochondria, 9 genes require intron splicing for the complete maturation of their respective transcripts, and 4 of these contain a single intron: *rps3*, *cox2*, *ccmFc* and *rpl2*. The others (*nad1*, *nad2*, *nad4*, *nad5* and *nad7*) contain multiple introns and are required for the normal function of mitochondrial complex I. Using the specific primer sets designed by Longevialle *et al*. (2007), we examined individual splicing events in mitochondria obtained from wild-type, *mterf15* and complemented plants. All splicing events showed similar results in both the wild-type and complemented plants; however, the RNA splicing of *nad2* intron 3 (*nad2-3*) was significantly reduced in *mterf15* plants (Figure 4A).

In Arabidopsis, the mature *nad2* mRNA is formed by merging exons from 2 different transcripts, *nad2a* and *nad2b*, through one *trans*-splicing and 3 *cis*-splicing events. The genomic fragment of *nad2a* carries exons 1 and 2 of *nad2* separated by *nad2* intron 1. The other 3 exons of *nad2*, exons 3 through 5, are from *nad2b* transcripts (Figure 4B). To validate the results of the qRT-PCR analysis, we used RT-PCR to examine the accumulation of un-spliced *nad2b* transcripts in *mterf15* plants; the amplification of *nad2* exon 5 by primers 3F and 3R was used for normalization (lower panel in Figure 4B). RT-PCR with primers 1F and 2R revealed no mature transcripts from the splicing of *nad2* intron 3 in *mterf15* plants (Figure 4B). Additionally, the transcript levels were greater in *mterf15* plants than in wild-type plants upon amplification of the exon–intron junction with the primer sets 1F&1R and 2F&2R. This *nad2* intron 3 splicing defect was further confirmed by northern blot analysis. The *nad2b* transcripts revealed by probes for *nad2* exon 3 and 5 showed no mature *nad2* mRNAs in *mterf15* plants (m in Figure 4C) and was accompanied by the overaccumulation of un-spliced transcripts (u in Figure 4C). A similar accumulation of un-spliced transcripts in *mterf15* was confirmed using probes specific to *nad2* intron 3 (u in Figure 4C). This splicing defect was fully rescued in complemented plants. Another *cis*-splicing transcript, *nad1* exon 3, was used as a positive control, with comparable results to those observed in qRT-PCR: the splicing was normal, but the accumulation of *nad1-3* was greater in *mterf15* plants than in wild-type and complemented plants (Figure 4C).

Because mTERF15 functions as an RNA-binding protein and is involved in *nad2* intron 3 splicing, mTERF15 may directly interact with *nad2* intron 3. First, we confirmed the interaction between mTERF15 and full-length *nad2* intron 3 (∼2.6 kb) by northwestern blot analysis (Figure 3, lowest panel). The interaction between mTERF15 and *nad2* intron 3 was stronger than the interaction with total RNA. We first examined the interaction between mTERF15 and full-length *nad2* intron 3 (∼2.6 kb) by northwestern blot analysis (Figure 3, lowest panel). The interaction between mTERF15 and *nad2* intron 3 was stronger than the interaction with total RNA. Then RNA-EMSA (RNA-Electrophoresis Mobility Shift Assay) was used to confirm the RNA-binding activity of mTERF15 (Figure S6). Additionally, two unrelated mitochondrial intron sequences, *ccmF_C_* and *rpl2*, were used as the control probes in RNA-EMSA. Since the full length of *nad2* intron 3 is too large to be used as the probe for RNA-EMSA, we divided it into five fragments (approximately 500 bp each), named Fragment 1-5 (Figure S6A). Fragment 1, part of domain 1, is the largest and substantially varies in sequence and secondary structure among characterized group II introns. Fragment 5 contains phylogenetically conserved and typical RNA structure found in all mitochondrial introns [51]. As shown in Figure S6B, only Fragment 1 could be recognized by mTERF15 in a dosage-dependent manner. Neither Fragments 2–5 nor *ccmFc* and *rpl2* could be recognized by mTERF15. Above results suggest that mTERF15 is an RNA-binding protein and is involved in the proper splicing of *nad2* intron 3.

**Figure 5:**
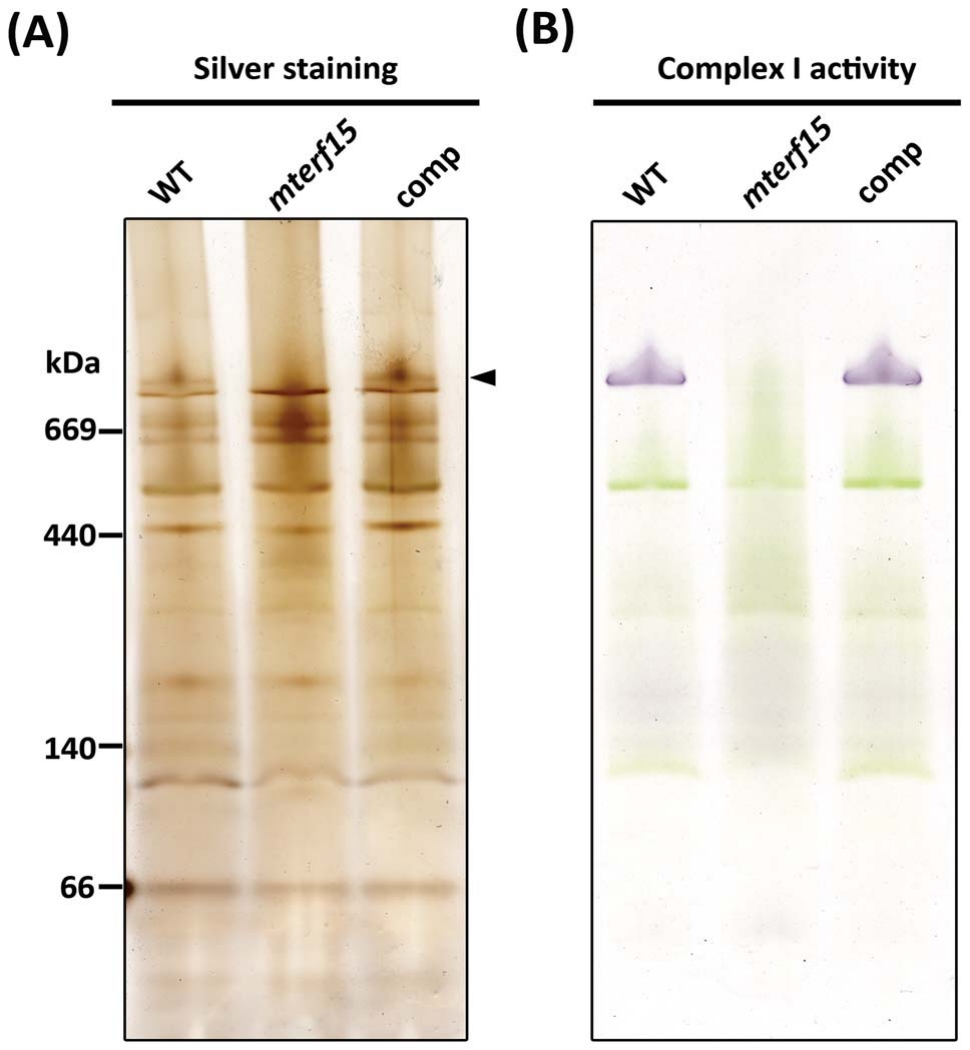
Clear native PAGE analysis of mitochondrial complex I activity. (**A**) Silver staining of the protein amount corresponding to crude mitochondria. (**B**) Complex I activity in crude mitochondria. Activity was visualized as a purple-blue color resulting from the utilization of the substrate (NADH) and the electron acceptor (nitroblue tetrazolium). Arrowhead indicated the band corresponding to Complex I.

In Arabidopsis, the *nad2* gene encodes a subunit of mitochondrial complex I (an NADH:ubiquinone oxidoreductase), which is required to translocate protons from the mitochondrial matrix into the intermembrane space. Complex I activity was therefore investigated in *mterf15*, in which the splicing of *nad2* intron 3 is defective. Isolated crude mitochondria were subjected to complex separation on clear native gels to visualize protein complexes by silver staining (Figure 5A) or to complex I activity assays (Figure 5B). The amount and activity of complex I were greatly reduced in *mterf15*, whereas the wild-type and complementation lines showed normal activity. Therefore, intron-splicing defects in *nad2* intron 3 led to reduced complex I formation and activity. Disturbing mitochondrial complex I impairs energy generation and leads to the extreme retardation of diverse aspects of growth and development in *mterf15* plants (Figure 1).

**Figure 6:**
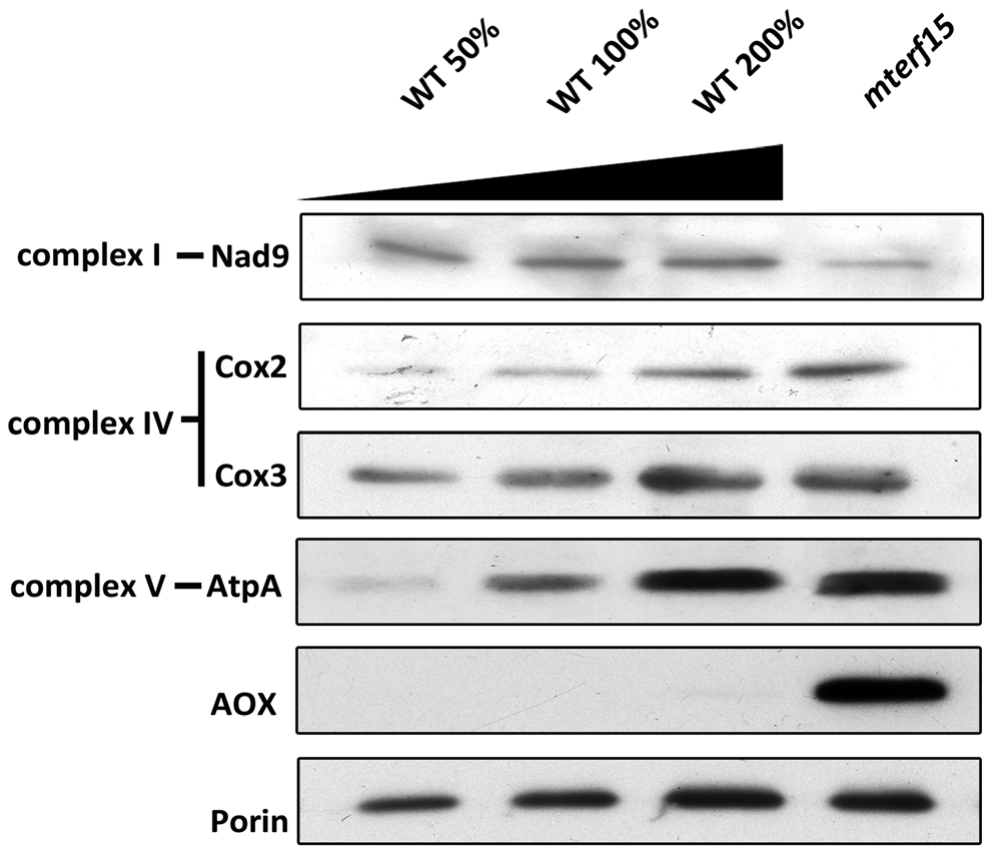
Relative accumulation of mitochondrial proteins in *mterf15* mutants. Crude mitochondria were isolated from the wild-type, *mterf15* and complementation lines. Following protein quantification based on the Bradford assay, equal amounts of protein were subjected to immunoblot analysis using various antibodies recognizing Nad9 [55], Cox2, Cox3, AtpA [56], and AOX [57], as well as Porin.

The steady-state levels of several mitochondrial proteins, including Nad9, Cox2, Cox3, AtpA, alternative oxidase (AOX) and the channel-forming protein Porin, were examined by immunoblot analysis of crude mitochondrial extracts from wild-type and *mterf15* plants (Figure 6). The steady-state levels of 3 proteins, Cox2, Cox3 and AtpA, were greater in *mterf15* plants than in wild-type plants. The level of alternative oxidase (AOX) was significantly increased in *mterf15* plants, which is consistent with a defect in NADH oxidation [46]. The level of another complex I subunit, Nad9, was decreased in *mterf15* plants, which may be due to an imbalance in the stoichiometry of multi-subunit complexes [47].

## Discussion

Our genetic, molecular and biochemical results indicate that mTERF15 is a unique, mitochondria-localized protein (Figures S2 and S4) with RNA-binding activity (Fig. 3). This protein is critical for post-transcriptional modification in the RNA splicing of mitochondrial *nad2* intron 3 (Figure 4). Mutating *mTERF15* impaired the normal activity of mitochondrial respiratory chain complex I (Figure 5), thereby resulting in abnormal mitochondrial development (Figure 2) and the widespread retardation of plant growth and development (Figure 1).

mTERFs constitute a broad family of eukaryotic proteins that are essential for the initiation and termination of organellar transcription and the translation and replication of the organellar transcription machinery [12]; however, mounting evidence suggests an emerging role for these proteins in RNA splicing in plants [39]. The human genome contains only 4 genes encoding mTERF paralogs, whereas the Arabidopsis genome contains at least 35 genes for mTERF proteins with a diverse arrangement and number of mTERF motifs. Using transient assays and stable transgenic plants expressing GFP-tagged mTERF proteins, Babiychuk *et al*. (2011) identified 11 and 17 mTERF proteins that localize to chloroplasts and mitochondria, respectively. However, only a few mTERFs have been well studied and are essential for vegetative growth [24]–[26] and embryogenesis [22], [23]. Whether Arabidopsis mTERF proteins share similar conserved molecular functions with their mammalian counterparts or have evolved additional regulatory mechanisms is unclear. In contrast to animal cells, plant cells harbor 2 types of nucleoid-containing organelles – chloroplasts and mitochondria. Moreover, plant mitochondrial genomes are much larger and more complex, requiring intron splicing for proper gene expression [48]. The biological functions of mTERF proteins may be complicated in plant cells because recent co-expression analyses of the 35 Arabidopsis mTERF members indicated the association of mTERF proteins with DNA and RNA metabolism [49]. Recently, it was found that Zm-mTERF4, an ortholog of Arabidopsis BSM/RUG2, is required for the splicing of several RNAs necessary for plastid translation in maize [39]. Therefore, an understanding of the mTERF family may provide new insights into the plant-specific functions of mTERF proteins in the transcriptional and post-transcriptional regulation of organellar nucleoids, as reported in this study.

In flowering plants, recombinogenic events such as the creation of intron-split genes greatly affect the dynamic nature of the mitochondrial genome [50]. The Arabidopsis mitochondrial genome contains 23 group II introns, and most are found dispersed in *nad* genes. The exons, including the flanking introns of these genes dispersed among the mtDNA, are transcribed and are the mRNAs generated by the splicing machinery. Until now, knowledge of the splicing machinery found in Arabidopsis mitochondria had been limited by the difficulty of organellar genome manipulation. However, a growing number of studies have revealed the involvement of a nuclear-encoded splicing factor in the excision of these introns [51]. Several proteins are involved in *nad2* intron splicing. For example, 2 proteins, ABA overly-sensitive 5 (ABO5) and RCC1/UVR8/GEF-like 3 (RUG3), were identified as splicing factors regulating the *cis*-splicing of *nad2* intron 3 [35], [52]. *ABO5*, which encodes a PPR protein, was isolated from a mutational screen for ABA sensitivity and is required for *cis*-splicing of *nad2* intron 3 in mitochondria [35]. Another protein, *RUG3*, which encodes an RCC1/UVR8-like protein, is responsible for the efficient splicing of *nad2* introns 2 and 3 in mitochondria [52]. The mTERF15 protein, identified in this study, is required for *nad2* intron 3 RNA splicing, as demonstrated by RT-PCR and by northern blotting showing the accumulation of *nad2* intron 3 in *mterf15* plants (Figure 4B–C). Moreover, RNA-binding assays also demonstrated the direct interaction between mTERF15 and *nad2* intron 3. Our results suggest that mTERF15 is a new splicing factor in mitochondria. The next steps are to identify the specific elements of *nad2* intron 3 that are required for the binding of mTERF15 and to explore the relationships and/or potential interactions between ABO5, RUG3 and mTERF15 in *nad2* intron 3 splicing.

The *nad2* intron 3 splicing defect led to the loss of mitochondrial complex I activity in *mterf15*. Similar results were observed with the *RUG3* mutant [52]. Both mutants showed reduced root growth, small stature, and growth retardation. Another complex I mutant, *ndufs4* (NADH dehydrogenase [ubiquinone] fragment S subunit 4), also exhibited a similar phenotype [53]. Although approximately one-third of mitochondrial ATP production is associated with complex I, the presence of alternative NADH dehydrogenases allows the electron transport chain to bypass complex I when complex I efficiency is reduced or when plants encounter stress [54]. Interestingly, *abo5*, *rug3* and *ndufs4* plants produced viable seeds and propagated to the next generation, whereas *mterf15* showed a more severe phenotype. Homozygous *mterf15* plants showed a similar phenotype as these mutants in terms of complex I deficiency; however, seeds from homozygous *mterf15* plants could not germinate, even in solid medium over three months. It is possible that mTERF15 in maternal tissues of heterozygotic *mterf15* embryo may partially contribute to the developing homozygous *mterf15* seeds, which is completely absent in the developing seeds of homozygous *mterf15* plants. This suggests that the germination defect of *mterf15* is not only due to embryo lethality but also caused by aberrant reproductive organs, especially maternal tissues. The defective maternal tissues may reduce the flow of nutrient into the endosperm and eventually cause seed lethality [58]. Therefore, mTERF15 may have additional roles in mitochondria, as observed in other eukaryotes, during embryogenesis and seed development.

Here, we identify mTERF15 as an RNA-binding protein that is required for *nad2* intron 3 splicing in mitochondria. The disruption of this splicing event leads to decreased complex I activity as well as growth and developmental retardation in *mterf15* mutants. Further studies of the detailed mechanism of mTERF15 are required to reveal the molecular mechanisms involved in post-transcription regulation in Arabidopsis mitochondria.

## Supporting Information

Figure S1 ***mTERF15p::mTERF15-GFP*** **fully complements developmental defects found in** ***mterf15*** **homozygous mutant.** 7-day-old seedlings from 6 independent complementation lines, homozygotes for *mTERF15* mutation and harbouring at least one copy of the transgenes *mTERF15p::mTERF15-GFP*. Seeds were surface-sterilized and germinated on half MS medium under 22°C with light period control (16-h light/8-h dark).
(TIF)

Figure S2 **Protein alignment and phylogenetic tree of mTERF15 and homologs.** (A) Protein sequence alignment of mTERF15 and its homologs in *Vitis vinifera* (Vv), *Populus trichocarpa* (Pt), *Brachypodium distachyon* (Bd), *Sorghum bicolor* (Sb) and *Zea mays* (Zm). The alignment was generated by use of CLUSTALW, which creates pairwise alignments to calculate the divergence between pairs of sequences. Five mTERF motifs present in all homologs are underlined in black. Asterisks indicate the location of highly conserved proline residues. (B) Phylogenetic tree of mTERF15 and its homologs. The phylogenetic tree was created with use of Molecular Evolutionary Genetics Analysis (MEGA) by the Unweighted Pair Group Method with Arithmatic Mean (UPGMA) method.
(TIF)

Figure S3 **Spatial expression profile of** ***mTERF15*** **.** qRT-PCR analysis of the expression of *mTERF15* in seedlings, roots, stems, rosette leaves, flowers, siliques and pollen. Primers used in this experiment are in Table S1.
(TIF)

Figure S4 **Subcellular localization of mTERF15 protein.** Confocal microscopy of the localization of the mTERF15 protein in root hairs of the *mterf15* complementation line harboring mTERF15p::mTERF15-GFP. (A) Signal corresponding to the mTERF15-GFP fusion protein; (B) signal corresponding to the MitoTracker marker; and (C) merged signals from GFP and the MitoTracker marker. Scale bar  = 10 µm.
(TIF)

Figure S5 **DNA-binding assay with mTERF15 recombinant protein.** (A) *In vitro* binding studies of dsDNA-cellulose with mTERF15-GST and GST. mTERF15-GST and GST were incubated with dsDNA-cellulose for 2 h at room temperature. The resin was washed with washing buffer 3 times and after different salt concentrations to elute bound protein. The detailed experimental procedure was previously described (Wobbe and Nixon, 2013). (B) Southwestern blot assay of DNA-binding ability by mTERF15-strep. The upper panel is Coomassie brilliant blue (CBB) staining of MBP-strep and mTERF15-strep without the first N-terminal 34 amino acid. The lower panel is sourthwestern blot analysis with radioisotope-labeled HindIII-digested mtDNA. **Methods of Figure S5: DNA-binding assay with dsDNA-cellulose resin.** This experimental procedure has been described previously (Wobbe & Nixon, 2013). Briefly, 1.8 µM of the mTERF15-GST fusion protein and GST control were incubated with 60 mg dsDNA-cellulose resin (Sigma) in DCBB buffer [50 mM HEPES, pH 8.0, 50 mM NaCl, 1 mM EDTA, 1 mM beta-mercaptoethanol and protease inhibitor cocktail (Roche)] at room temperature for 2 hr and under gentle rotation. The resin was washed 3 times with DCBB buffer, then bound protein was eluted at different salt concentrations (100, 300, 600 and 1000 mM NaCl in DCBB buffer). The samples corresponding to each step were collected and analyzed on SDS-PAGE. **Southwestern blot assay.** The recombinant protein were separated on 12% SDS-polyacrylamide gels and transferred onto a PVDF membrane and then renatured overnight in renaturation buffer (0.1 M Tris-HCl, pH 7.5 and 0.1% (v/v) NP-40) at 4°C. After 4 washes with renaturation buffer (15 min), blots were blocked with blocking buffer [(10 mM Tris-HCl, pH 7.5, 2 mM DTT, 5% (w/v) BSA and 0.01% (v/v) Triton X-100)] at room temperature for 5 min. Mitochondrial DNA was isolated from crude mitochondria fraction, digested with HindIII and labeled with radioisotope using T4 polynucleotide kinase (Fermentas). Hybridization was performed at 4°C overnight in the presence of 5 µg labeled mtDNA. Blots were washed 4 times (5 min) in washing buffer (10 mM Tris-HCl, pH 7.5, 2 mM DTT) to remove unbound DNA. Radioisotope signals from PVDF membrane were detected via autoradiography on X-ray film (Kodak).
(TIF)

Figure S6 **RNA-EMSA of mTERF15 recombinant protein with different mitochondrial transcribed intron fragments.** (A) The diagram of *nad2* intron 3 and the regions and sizes of 5 fragments in *nad2* intron 3 used for assays. (B) Different concentration mTERF15 recombinant proteins (20, 10, 5 and 0 nM) were incubated with 100 nM radioisotope labeled indicated RNA probes. **Methods of Figure S6:** mTERF15 fusion protein were purified as mentioned in Northwestern blot analysis. For *in vitro* transcription, mitochondrial introns were amplified and cloned to pJET vector. Primer sets are nad2int3-1F “ACGCCAAGCTATTTAGGTGACACTATAGAATACGGGCGGCTGTAGGACGGAC” and nad2int3-1R “CTGTTCACCGTTGGATCTCGCC” for *nad2* intron 3 Fragment 1; nad2int3-5F “ACGCCAAGCTATTTAGGTGACACTATAGAATACGCGTGTTATCTGAAGGGAGCACG” and nad2int3-5R “GGGGGAGGGGGTTTTCTTCG” for *nad2* intron 3 Fragment 5; rpl2-F “ACGCCAAGCTATTTAGGTGACACTATAGAATACATGAGACCAGGGAGAGCAAGAGCAC” and rpl2-mid-intron-R “CGTTGCTAAGCCAAGGTCCC” for *rpl2* intron; ccmFc-F “ACGCCAAGCTATTTAGGTGACACTATAGAATACATGGTCCAACTACATAACTTTTTC” and ccmFc-mid-intron-R “GCTTTGCCAACACAACATTAGG” for *ccmF_C_* intron. Then, RNA probes were *in vitro* synthesized using SP6 polymerase (Promega) and labeled with γ^32^P ATP using T4 polynucleotide kinase (Fermentas). Different concentration of mTERF15 recombinant proteins (20, 10, 5 and 0 nM) were incubated with 100 nM *in vitro* synthesized RNAs for 30 min at room temperature in binding buffer (20 mM Tris/HCl pH 7.5, 180 mM NaCl, 2 mM dithiothreitol, 17 µg/µl BSA, 0.5 mM EDTA, and 20 µg/ml heparin). The mixtures were separated by 5% polyacrylamide gel and gel was dried. Signals were detected via autoradiography on X-ray film (Kodak).
(TIF)

Table S1 **Primer sequences used in mTERF125 cloning, gene expression, and probe preparation for northern blotting and RT-PCR for the** ***nad2*** **exon–intron junction.**
(DOCX)

Table S2 **Primer sequences for determining RNA splicing efficiency in mitochondria.**
(DOCX)

Table S3 **Protein identity and similarity of mTERF15 with its plant homologs and mTERF1 in metazoans.**
(DOCX)
