# Supplementary figures and images for: Arabidopsis mTERF15 Is Required for Mitochondrial nad2 Intron 3 Splicing and Functional Complex I Activity
Source: PLoS One. 2014 Nov 17;9(11):e112360. doi: 10.1371/journal.pone.0112360 (PMC4234379; doi:10.1371/journal.pone.0112360)

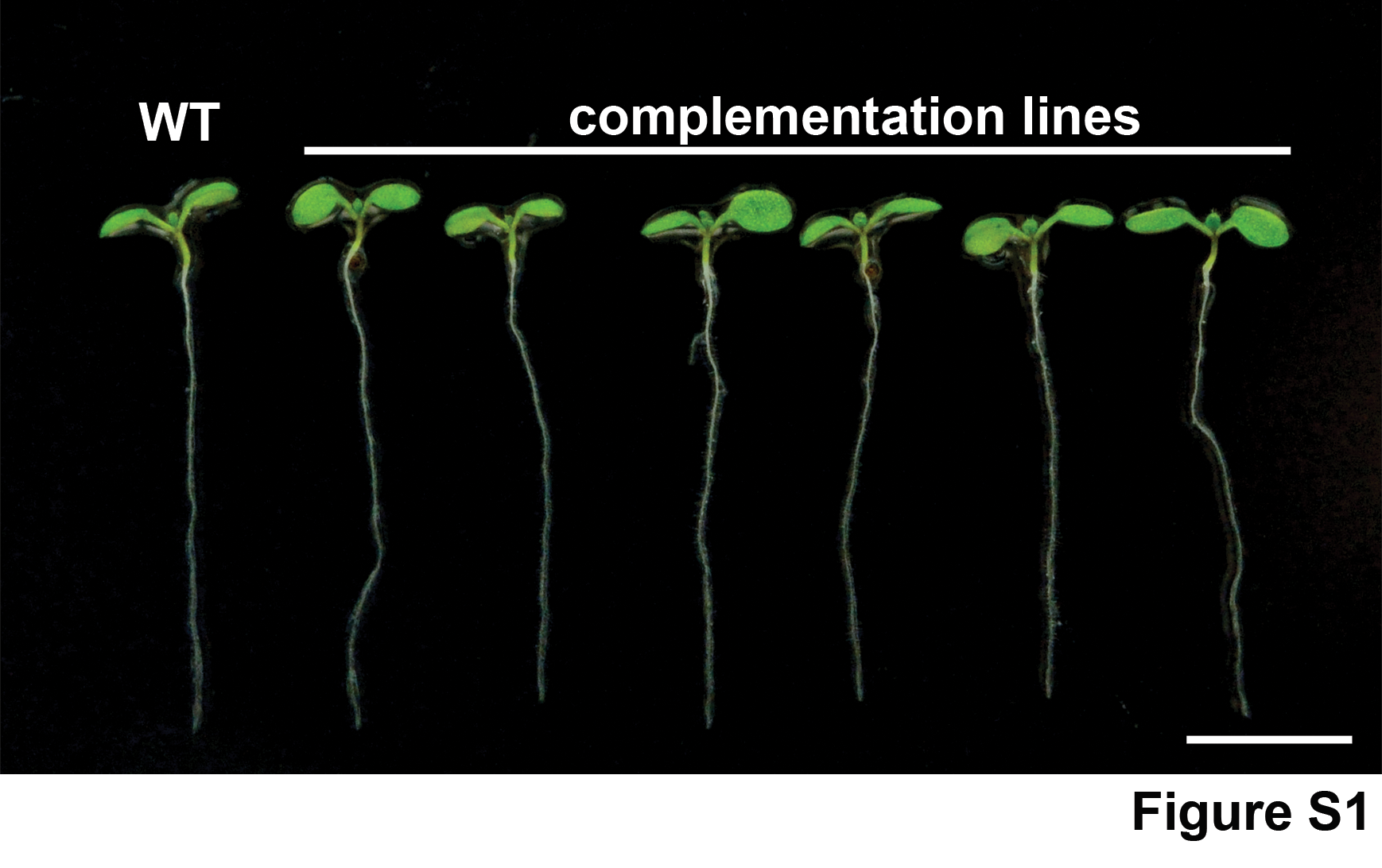

Supplement: Figure S1 — mTERF15p::mTERF15-GFP fully complements developmental defects found in mterf15 homozygous mutant. 7-day-old seedlings from 6 independent complementation lines, homozygotes for mTERF15 mutation and harbouring at least one copy of the transgenes mTERF15p::mTERF15-GFP. Seeds were surface-sterilized and germinated on half MS medium under 22°C with light period control (16-h light/8-h dark). (TIF) [file pone.0112360.s001.tif]

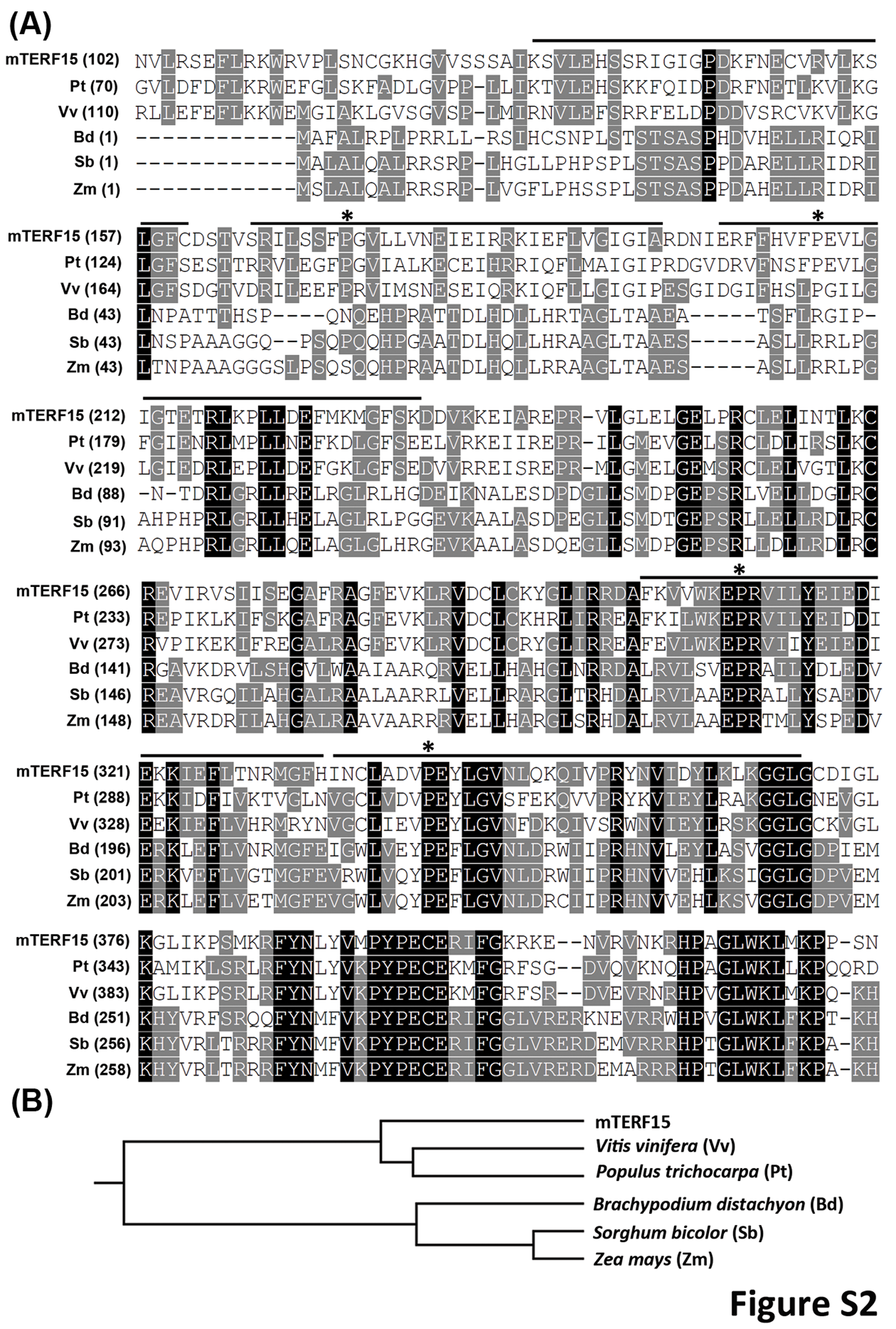

Supplement: Figure S2 — Protein alignment and phylogenetic tree of mTERF15 and homologs. (A) Protein sequence alignment of mTERF15 and its homologs in Vitis vinifera (Vv), Populus trichocarpa (Pt), Brachypodium distachyon (Bd), Sorghum bicolor (Sb) and Zea mays (Zm). The alignment was generated by use of CLUSTALW, which creates pairwise alignments to calculate the divergence between pairs of sequences. Five mTERF motifs present in all homologs are underlined in black. Asterisks indicate the location of highly conserved proline residues. (B) Phylogenetic tree of mTERF15 and its homologs. The phylogenetic tree was created with use of Molecular Evolutionary Genetics Analysis (MEGA) by the Unweighted Pair Group Method with Arithmatic Mean (UPGMA) method. (TIF) [file pone.0112360.s002.tif]

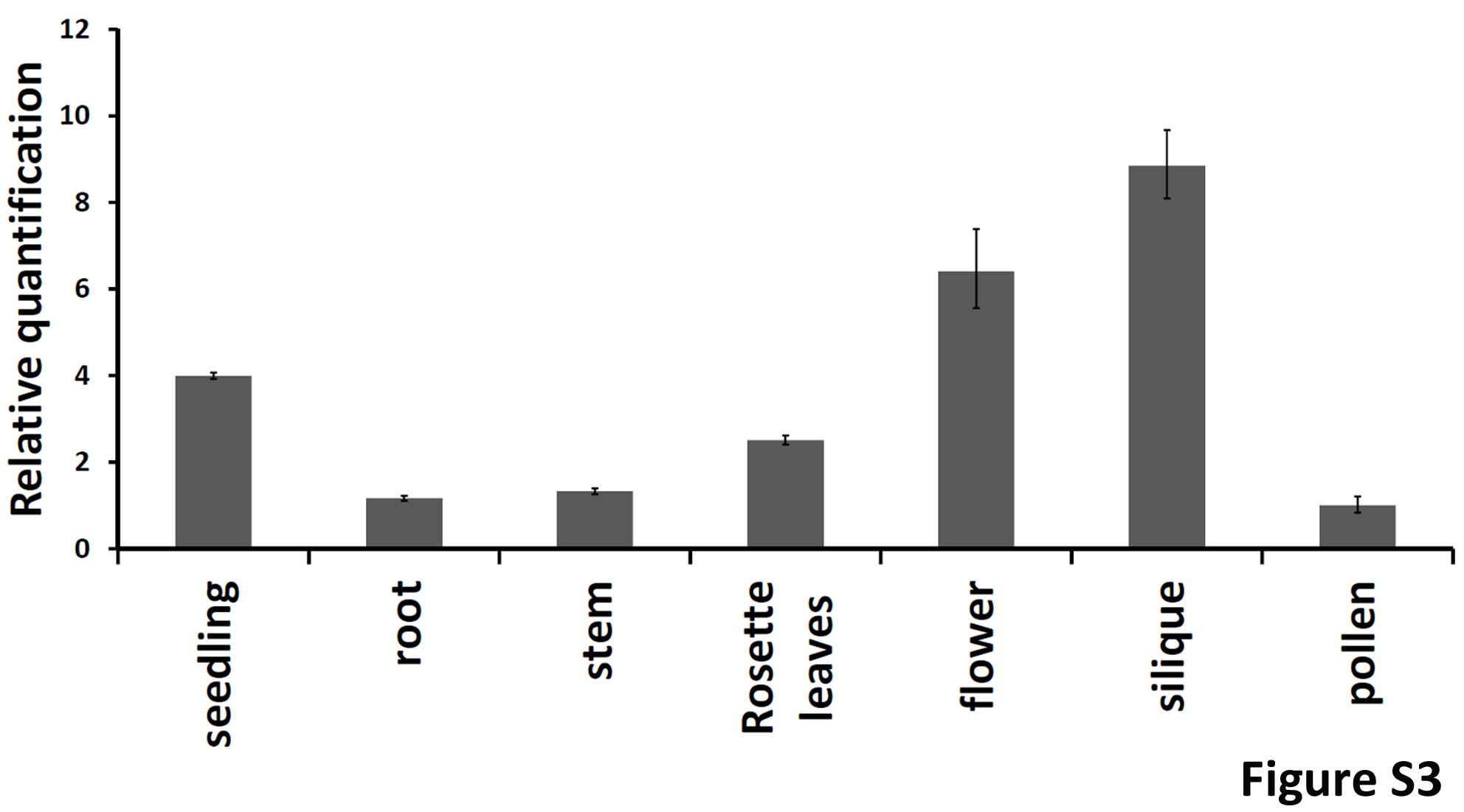

Supplement: Figure S3 — Spatial expression profile of mTERF15 . qRT-PCR analysis of the expression of mTERF15 in seedlings, roots, stems, rosette leaves, flowers, siliques and pollen. Primers used in this experiment are in Table S1. (TIF) [file pone.0112360.s003.tif]

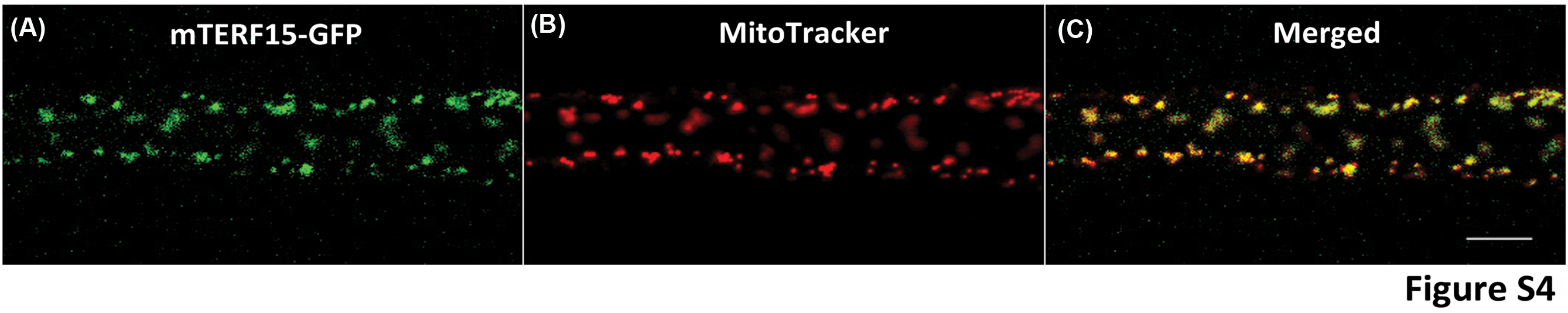

Supplement: Figure S4 — Subcellular localization of mTERF15 protein. Confocal microscopy of the localization of the mTERF15 protein in root hairs of the mterf15 complementation line harboring mTERF15p::mTERF15-GFP. (A) Signal corresponding to the mTERF15-GFP fusion protein; (B) signal corresponding to the MitoTracker marker; and (C) merged signals from GFP and the MitoTracker marker. Scale bar = 10 µm. (TIF) [file pone.0112360.s004.tif]

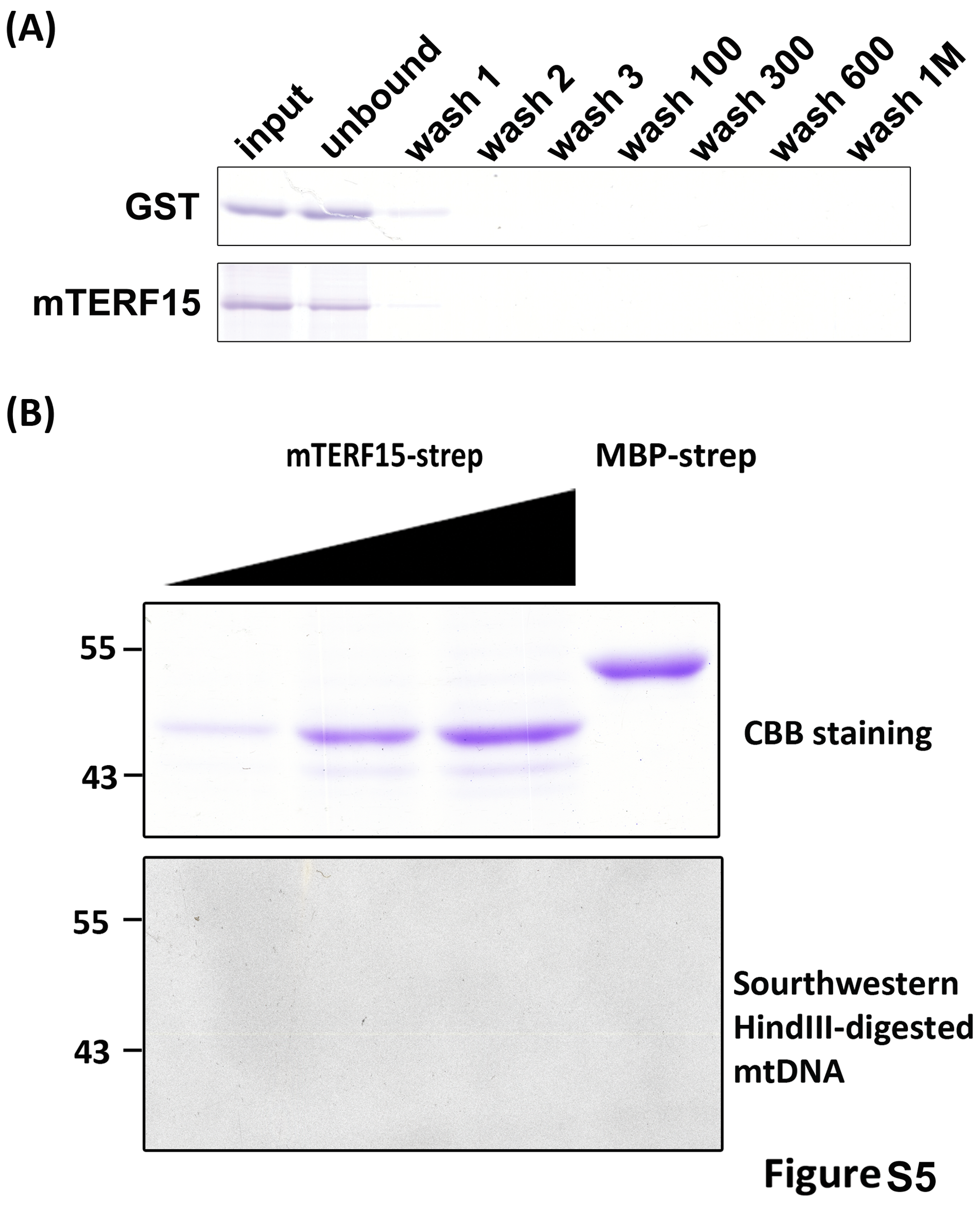

Supplement: Figure S5 — DNA-binding assay with mTERF15 recombinant protein. (A) In vitro binding studies of dsDNA-cellulose with mTERF15-GST and GST. mTERF15-GST and GST were incubated with dsDNA-cellulose for 2 h at room temperature. The resin was washed with washing buffer 3 times and after different salt concentrations to elute bound protein. The detailed experimental procedure was previously described (Wobbe and Nixon, 2013). (B) Southwestern blot assay of DNA-binding ability by mTERF15-strep. The upper panel is Coomassie brilliant blue (CBB) staining of MBP-strep and mTERF15-strep without the first N-terminal 34 amino acid. The lower panel is sourthwestern blot analysis with radioisotope-labeled HindIII-digested mtDNA. Methods of Figure S5: DNA-binding assay with dsDNA-cellulose resin. This experimental procedure has been described previously (Wobbe & Nixon, 2013). Briefly, 1.8 µM of the mTERF15-GST fusion protein and GST control were incubated with 60 mg dsDNA-cellulose resin (Sigma) in DCBB buffer [50 mM HEPES, pH 8.0, 50 mM NaCl, 1 mM EDTA, 1 mM beta-mercaptoethanol and protease inhibitor cocktail (Roche)] at room temperature for 2 hr and under gentle rotation. The resin was washed 3 times with DCBB buffer, then bound protein was eluted at different salt concentrations (100, 300, 600 and 1000 mM NaCl in DCBB buffer). The samples corresponding to each step were collected and analyzed on SDS-PAGE. Southwestern blot assay. The recombinant protein were separated on 12% SDS-polyacrylamide gels and transferred onto a PVDF membrane and then renatured overnight in renaturation buffer (0.1 M Tris-HCl, pH 7.5 and 0.1% (v/v) NP-40) at 4°C. After 4 washes with renaturation buffer (15 min), blots were blocked with blocking buffer [(10 mM Tris-HCl, pH 7.5, 2 mM DTT, 5% (w/v) BSA and 0.01% (v/v) Triton X-100)] at room temperature for 5 min. Mitochondrial DNA was isolated from crude mitochondria fraction, digested with HindIII and labeled with radioisotope using T4 polynucleotide kinase (Fermentas [file pone.0112360.s005.tif]

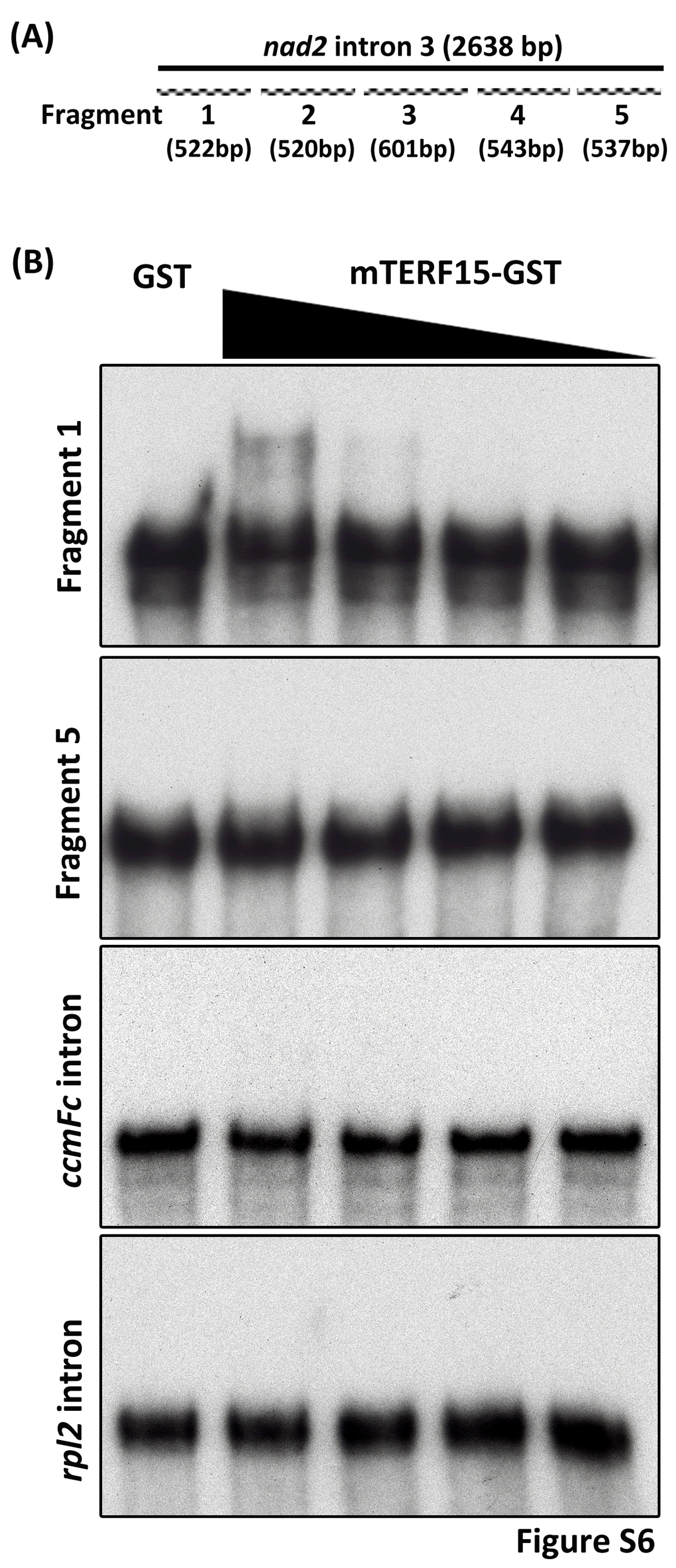

Supplement: Figure S6 — RNA-EMSA of mTERF15 recombinant protein with different mitochondrial transcribed intron fragments. (A) The diagram of nad2 intron 3 and the regions and sizes of 5 fragments in nad2 intron 3 used for assays. (B) Different concentration mTERF15 recombinant proteins (20, 10, 5 and 0 nM) were incubated with 100 nM radioisotope labeled indicated RNA probes. Methods of Figure S6: mTERF15 fusion protein were purified as mentioned in Northwestern blot analysis. For in vitro transcription, mitochondrial introns were amplified and cloned to pJET vector. Primer sets are nad2int3-1F “ACGCCAAGCTATTTAGGTGACACTATAGAATACGGGCGGCTGTAGGACGGAC” and nad2int3-1R “CTGTTCACCGTTGGATCTCGCC” for nad2 intron 3 Fragment 1; nad2int3-5F “ACGCCAAGCTATTTAGGTGACACTATAGAATACGCGTGTTATCTGAAGGGAGCACG” and nad2int3-5R “GGGGGAGGGGGTTTTCTTCG” for nad2 intron 3 Fragment 5; rpl2-F “ACGCCAAGCTATTTAGGTGACACTATAGAATACATGAGACCAGGGAGAGCAAGAGCAC” and rpl2-mid-intron-R “CGTTGCTAAGCCAAGGTCCC” for rpl2 intron; ccmFc-F “ACGCCAAGCTATTTAGGTGACACTATAGAATACATGGTCCAACTACATAACTTTTTC” and ccmFc-mid-intron-R “GCTTTGCCAACACAACATTAGG” for ccmFC intron. Then, RNA probes were in vitro synthesized using SP6 polymerase (Promega) and labeled with γ32P ATP using T4 polynucleotide kinase (Fermentas). Different concentration of mTERF15 recombinant proteins (20, 10, 5 and 0 nM) were incubated with 100 nM in vitro synthesized RNAs for 30 min at room temperature in binding buffer (20 mM Tris/HCl pH 7.5, 180 mM NaCl, 2 mM dithiothreitol, 17 µg/µl BSA, 0.5 mM EDTA, and 20 µg/ml heparin). The mixtures were separated by 5% polyacrylamide gel and gel was dried. Signals were detected via autoradiography on X-ray film (Kodak). (TIF) [file pone.0112360.s006.tif]
