# Supplementary material for: Arabidopsis mTERF15 Is Required for Mitochondrial nad2 Intron 3 Splicing and Functional Complex I Activity
Source: PLoS One. 2014 Nov 17;9(11):e112360. doi: 10.1371/journal.pone.0112360 (PMC4234379; doi:10.1371/journal.pone.0112360)
Supplement: Table S1 — Primer sequences used in mTERF125 cloning, gene expression, and probe preparation for northern blotting and RT-PCR for the nad2 exon–intron junction. (DOCX) [file pone.0112360.s007.docx]

**Table S1. Primer sequences used for cloning, gene expression and probe preparation for northern blot and RT-PCR analysis for *nad2* exon–intron junction.**

| Primer name | Sequence (5’ to 3’ orientation) |
| --- | --- |
| mTERF15 gDNA XmaI F | CCCGGGGATTAACCAATAACAAAGGTAAAGCATTTCGTCTAAACC |
| mTERF15 ORF SalI R | GTCGACAGCAAGTGACTCTATAAAGGACTTCATGTTCTG |
| mTERF15 complementary primer R | CCATATCAATTCAGAGCAGCTCATAGC |
| mTERF15 cDNA-R (XmaI) | ccccccgggAGCAAGTGACTCTATAAAGGACTTCATG |
| mTERF15 qPCR-F | GCCATACCCAGAGTGTGAAAGAATC |
| mTERF15 qPCR-R | CGATAGTCACTGAAAGTTCCTGATAACC |
| nad2 ex5-F | CAGTGGGAGTAGTGACTAGCGTTATAGG |
| nad2 ex5-R | TTAAAGATATGAACTGAGTGCCATTTGATG |
| nad2 ex3-F | CTATGGGTCTACTGGAGCTACCC |
| nad2 ex3-R | CTTGAATAGGAATCCTACAGCGATAG |
| nad2 int3-F | CCAAGTTCTGATAAGGAAGGAACAACC |
| nad2 int3-R | CTCTCTTTTCTAGTAGATGCCGAACCTG |
| nad1 ex3-F | CTGTACTAATATGTGTAGGTCCCCGTAATTC |
| nad1 ex3-R | GCCTGCAACTGATTCAGCTTCC |
| 1F | CTATGGGTCTACTGGAGCTACCC |
| 1R | GCTATGGTCTCAATGCCCTTAC |
| 2F | AGTTACAGGAACAGCCGTCTG |
| 2R | GCGCAATAGAAAGGAATGCT |
| 3F | CAGTGGGAGTAGTGACTAGCGTTATAGG |
| 3R | TTAAAGATATGAACTGAGTGCCATTTGATG |
| Actin1-F | CATCAGGAAGGACTTGTACGG |
| Actin1-R | GATGGACCTGACTCGTCATAC |
