# Supplementary material for: Arabidopsis mTERF15 Is Required for Mitochondrial nad2 Intron 3 Splicing and Functional Complex I Activity
Source: PLoS One. 2014 Nov 17;9(11):e112360. doi: 10.1371/journal.pone.0112360 (PMC4234379; doi:10.1371/journal.pone.0112360)
Supplement: Table S2 — Primer sequences for determining RNA splicing efficiency in mitochondria. (DOCX) [file pone.0112360.s008.docx]

**Table S2. Primer sequences used for determining RNA splicing efficiency in mitochondria.** Primers were designed according to Longevialle *et al.*, 2007.

| Primer name | Sequence (5’ to 3’ orientation) |
| --- | --- |
| rpl2-F | CCGAAGACGGATCAAGGTAA |
| rpl2-R | CGCAATTCATCACCATTTTG |
| rps3-F | AGCCGAAGGTGAGTCTCGTA |
| rps3-R | CCGATTTCGGTAAGACTTGG |
| cox2-F | TGGGGGATTAATTGATTGGA |
| cox2-R | TGATGCTGTACCTGGTCGTT |
| ccb452-F | GTGGGTCCATGTAAATGATCG |
| ccb452-R | CACATGGAGGAGTGTGCATC |
| nad1exon1-F | GACCAATAGATACTTCATAAGAGACCA |
| nad1exon5-R | TCTTCAATGGGGTCTGCTC |
| nad2exon1-F | GCGAGCAGAAGCAAGGTTAT |
| nad2exon5-R | TATTTGTTCTTCGCCGCTTT |
| nad4exon1-F | ATTCTATGTTTTTCCCGAAAGC |
| nad4exon4-R | TGAAATTTGCCATGTTGCAC |
| nad5exon1-F | TGGACCAAGCTACTTATGGATG |
| nad5exon5-R | GTTCCTGCGTTTCGGATATG |
| nad7exon1-F | ACCTCAACATCCTGCTGCTC |
| nad7exon5-R | AGGTGCTTCAACTGCGGTAT |
| nad2-R2 | GGGTTGTGGGGCTTACTTTC |
| nad1-1R | TTGCCATATCTTCGCTAGGTG |
| nad1-2F | ATTCAGCTTCCGCTTCTGG |
| nad1-2R | TCTGCAGCTCAAATGGTCTC |
| nad1-3F | AAAAGAGCAGACCCCATTGA |
| nad1-3R | TCCGTTTGATCTCCCAGAAG |
| nad1-4F | AGCCCGGGATCTTCTTGA |
| nad2-1R | GGATCCTCCCACACATGTTC |
| nad2-2F | AAAGGAACTGCAGTGATCTTGA |
| nad2-2R | AATATTTGATCTTAGGTGCATTTTC |
| nad2-3F | GCGCAATAGAAAGGAATGCT |
| nad2-3R | CTATGGGTCTACTGGAGCTACCC |
| nad2-4F | CAAAGGAGAGGGGTATAGCAA |
| nad4-1R | GAAAAACTGATATGCTGCCTTG |
| nad4-2F | AATACCCATGTTTCCCGAAG |
| nad4-2R | TGCTACCTCCAATTCCCTGT |
| nad4-3F | TTCCTCCATAAATTCTCCGATT |
| nad5-1R | CCATGGATCTCATCGGAAAT |
| nad5-2F | AACTCGGATTCGGCAAGAA |
| nad5-2R | CTGGCTCTCGGGAGTCTCTT |
| nad5-3F | AACTCGGATTCGGCAAGAA |
| nad5-3R | CTGGCTCTCGGGAGTCTCTT |
| nad5-4F | AACATTGCAAAGGCATAATGA |
| nad7-1R | AAGGTAAAGCTTGAAGATAAGTTTTGT |
| nad7-2F | GAGGGACTGAGAAATTAATAGAGTACA |
| nad7-2R | TGGTACCTCGCAATTCAAAA |
| nad7-3F | ACTGTCACTGCACAGCAAGC |
| nad7-3R | CATTGCACAATGATCCGAAG |
| nad7-4F | GATCAAAGCCGATGATCGTAA |
