# Supplementary material for: Arabidopsis mTERF15 Is Required for Mitochondrial nad2 Intron 3 Splicing and Functional Complex I Activity
Source: PLoS One. 2014 Nov 17;9(11):e112360. doi: 10.1371/journal.pone.0112360 (PMC4234379; doi:10.1371/journal.pone.0112360)
Supplement: Table S3 — Protein identity and similarity of mTERF15 with its plant homologs and mTERF1 in metazoans. (DOCX) [file pone.0112360.s009.docx]

**Table S3. Comparisons of protein identity and similarity of mTERF15 with its plant homologs and mTERF1 in metazoans.**

| Accession | Species | Protein length (aa) | Identity  (%) | Similarity (%) | |
| --- | --- | --- | --- | --- | --- |
| **mTERF15 orthologs in flowering plants** | | | | |  |
| NP_565080.1 | *A. thaliana* | 445 | - | - | |
| XP_002324772.1 | *Populus trichocarpa* | 412 | 63.28 | 71.31 | |
| XP_002265430.1 | *Vitis vinifera* | 451 | 63.47 | 72.08 | |
| XP_003575506.1 | *Brachypodium distachyon* | 387 | 25.04 | 33.84 | |
| XP_002442362.1 | *Sorghum bicolor* | 327 | 36.71 | 45.88 | |
| NP_001144077.1 | *Zea mays* | 329 | 36.9 | 46.27 | |
| **mTERF1 in metazoans** | | | | |  |
| NP_001288063.1 | *Homo sapiens* | 379 | 12.05 | 23.21 | |
| NP_001013041.2 | *Mus musculus* | 379 | 12.05 | 23.21 | |
| NP_445951.1 | *Rattus norvegicus* | 374 | 11.16 | 23.43 | |
| XP_003200550.1 | *Danio rerio* | 366 | 11.6 | 24.77 | |

Protein sequence were obtained from NCBI database and aligned by use of CLUSTALW, which creates pairwise alignments to calculate the divergence between pairs of sequences. The identity and similarity were calculated by SIAS database (<http://imed.med.ucm.es/Tools/sias.html>).
